# Supplementary material for: Rapid Exploration of the Assembly Chemical Space of Molecular Graphs
Source: J Chem Inf Model. 2025 Dec 7;65(24):13203–14. doi: 10.1021/acs.jcim.5c01964 (PMC12728931; doi:10.1021/acs.jcim.5c01964)
Supplement: Supplementary file 1 [file ci5c01964_si_001.pdf]

1Supplementary Information for

Rapid Exploration of Assembly Chemical Space of Molecular Graphs

Ian Seet[1], Keith Y. Patarroyo[1], Gage Siebert[3], Sara I. Walker[2,3], Leroy Cronin[1]*

[1]School of Chemistry, University of Glasgow, Glasgow, G12 8QQ, UK.

[2]BEYOND Center for Fundamental Concepts in Science, Arizona State University, Tempe, AZ, USA

[3]School of Earth and Space Exploration, Arizona State University, Tempe, AZ, USA

*Corresponding author email: Lee.Cronin@glasgow.ac.uk

Contents

1. Pathway Reconstruction 1

2. COCONUT Database Computation 5

3. Calculation of the Tanimoto similarity metrics with ECFP fingerprints and the MCS similarity scores 6

References 6

1. Pathway Reconstruction

The assembly index algorithm described in this paper uses the technique of iteratively fragment the original molecule and generate as a result a set of duplicate structures and a remnant structure, Figure 2. Here we describe a general procedure to reconstruct an assembly pathway that works for any assembly index algorithm whose output is in the specified duplicate and remnant structures format.

First we introduce a bag of *pieces*, this bag is a list of graph fragments that will contain remnant graphs and constructed graphs. Now the idea is that we are going to build each of the *duplicates* in order of size step by step using the *remnant* as building blocks. After creating a single duplicate, it is replicated, and both graphs are added to the bag of pieces while r1emoving the pieces used to construct the original

duplicate. When we have generated all duplicates, we are going to join all the pieces from the bag, i.e. the duplicates together with what remains from the remnant.

In detail this is described in Algorithm A1. We initialize the pieces with the remnant structure and sort the duplicate structures. We sort the duplicate structures since some duplicate structures need previous smaller duplicate structures to be constructed before them, Figure 2. Then we start building duplicates from pieces in the bag, the smallest duplicate is constructed piece by piece and then is replicated, both pieces are added to the bag. Note that the duplicate and its precise replication in the original molecular graph are given as output from the algorithm. This is done until we build all the duplicates. If a duplicate had already been constructed, then its replicated once and this replicated piece is added to the bag. Note that in the bag some remnant pieces might not have been used. The final step is to join the compound pieces from the pieces bag one by one. In a similar way as before, when we join two pieces from the bag, we'll add one piece and remove the components. This is continued until we get the original graph or until the pieces of the bag cannot be connected anymore(in the case of joint assembly spaces). At each joining operation we store the resulting structure in a steps list in order to store the construction steps.

In the Figure A1 we provide an example of a construction process for the remnant and duplicate graphs coming from the iterative fragmentation of the molecule in Figure 2. We start by building the smallest duplicate with one joining operation and then it is duplicated, both of this pieces are added to the pieces bag, while the original pieces to construct it are deleted. Next we do the same with the next duplicate, note that in order to build this duplicate we need two joining operations, represented by a nought and a cross. Again, after the duplication these two pieces are added to the bag and its original parts are deleted. Finally we join each piece one by one, resulting in two joining operations to generate the original molecule. In this procedure we performed five joining operations, verifying that the assembly index of this molecule is $a_i = 5$

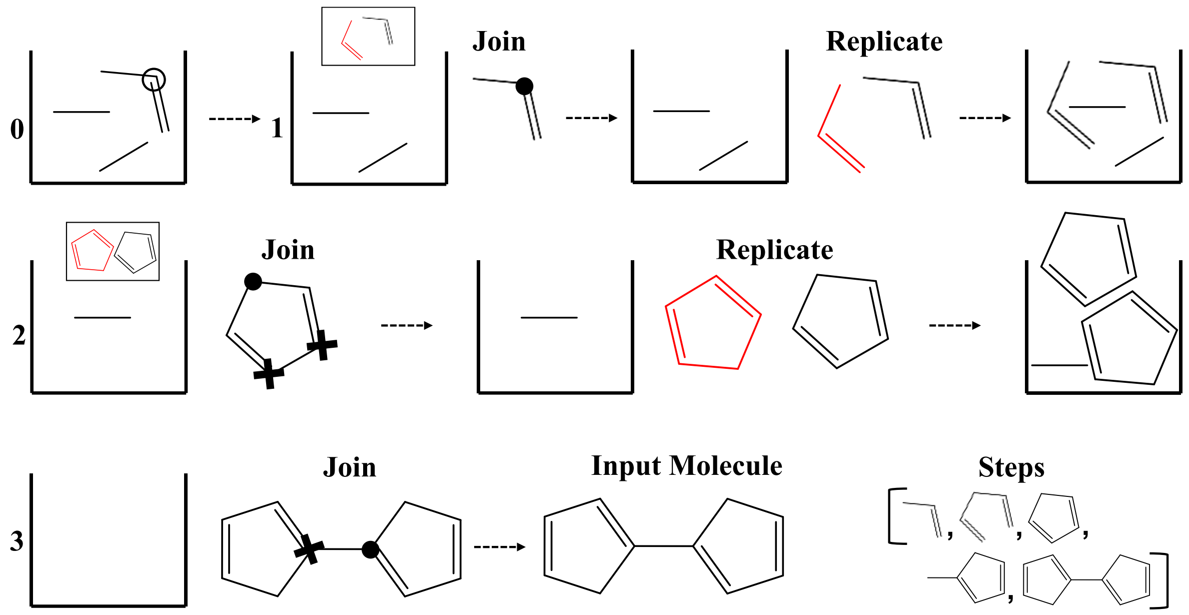

Fig A1: Reconstruction of the molecule in Figure 2 given the remnant and duplicate structures found in the last step of the iterative searching of the assembly index algorithm. Note that, the connected parts of the remnant structure in the initial pieces bag should also be constructed from the building blocks.

With very simple modifications to this algorithm we can store the building blocks or the steps that were used to build a given step. If we store this connectivity information in a Directed Acyclic Graph (DAG), as described in the section on the algorithm, we would obtain the assembly space of the given molecular graph or set of molecular graphs. In the main text, we show three such assembly spaces, Figure 1, the assembly space of the molecule benzoic acid, Figure 4 ,the assembly space of the molecule Taxol and Figure 6, the joint assembly space of all 20 standard amino acids. These graphs were produced with Mathematia 14, from the DAG.

```
Function Consistent_Join (Pieces, Steps):
        for p ∈ Pieces do
            for p̂ ∈ Pieces do
                for v ∈ p̂ do
                    if v ∈ p̂ ∧ p ≠ p̂ then
                        Steps.append(p̂ ⊙ p)
                        Pieces.append(p̂ ⊙ p)
                        Pieces.remove([p̂, p])

                    end
                end
            end
        end

Function Duplicate_Construction (Duplicates, Pieces, Steps):
        for d ∈ Duplicates do
            if d[1] ∈ Pieces then
                    Pieces.append(d[0])
            else
                    PiecesFiltered:=Filter_Pieces(Pieces,d[1])
                    Pieces.remove(PiecesFiltered)
                    while PiecesFiltered.size()≠ 1 do
                            Consistent_Join(PiecesFiltered,Steps)
                    end
                    Pieces.append({d[0],PiecesFiltered[0]})
            end
            Duplicates.remove(d)
        end

Function Generate_Pathway (Duplicates,Remnants):
      Steps:=[]
      Sort(Duplicates)
      Pieces:=Remnants
      Duplicate_Construction(Duplicates,Pieces)
      active :=true
      while active==true do
            LenBefore:=Pieces.size()
            Consistent_Join(Pieces,Steps)
            if LenBefore==Pieces.size() then
                    active :=false
            end
      end
return Steps
```

Algorithm A1: Pathway reconstruction.

2. COCONUT Database Computation

We proceeded to download the COCONUT database[1], then classified the molecules in order of the number of bonds. Then, choose the molecules with fewer than 60 bonds and divide them into 100 unique sets. We used our local server to compute in parallel about 20 sets at a time, evaluating the assembly index of molecules until convergence. We measured the computation time and the memory that each of the processes took to compute the assembly index until convergence. We checked that the behavior of the assembly index lies in the correct regions of linear and logarithmic behavior depending on bonds, Figure A2. Also, this is the behavior that was obtained by previous methods[2].

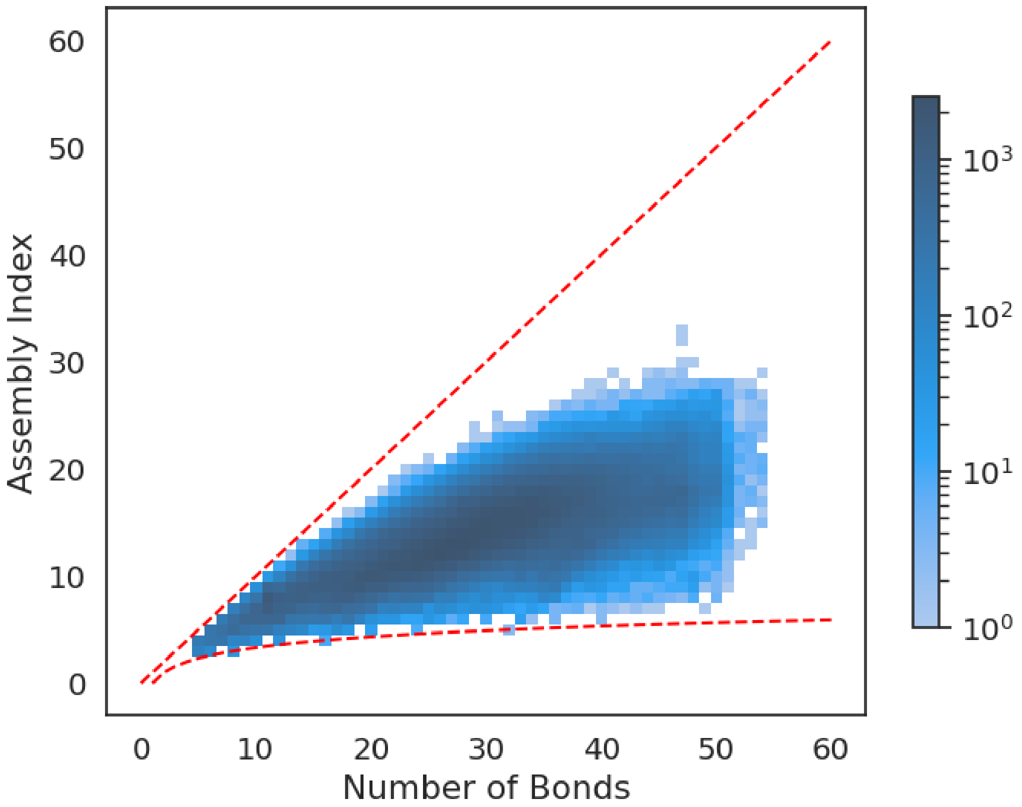

Fig A2: Distribution of assembly index against the number of bonds, based on 300k structures sampled from the COCONUT Database. The theoretical upper limit is linear, and the lower limit is logarithmic in the number of bonds. The assembly index values were calculated using the exact algorithm described in this paper.

Then we used Python and Seaborn to plot the behavior of the assembly index against the amount of memory and the computational time, and also did a contour plot depending on the number of bonds, see main text Figure 7.

3. Calculation of the Tanimoto similarity metrics with ECFP fingerprints and the MCS similarity scores

The Tanimoto similarity with ECFP-4 and ECFP-6 fingerprints and the MCS score were calculated using the RDKit[3] functions GetMorganFingerprintAsBitVect, TanimotoSimilarity and FindMCS. For the MCS score, the ringMatchesRingOnly and completeRingsOnly parameters were set to True.

References

1. Sorokina, M., Merseburger, P., Rajan, K., Yirik, M. A. & Steinbeck, C. COCONUT online: Collection of Open Natural Products database. *J. Cheminformatics* **13**, 2 (2021).
2. Jirasek, M. *et al.* Investigating and Quantifying Molecular Complexity Using Assembly Theory and Spectroscopy. *ACS Cent. Sci.* **10**, 1054–1064 (2024).
3. Greg Landrum *et al.* rdkit/rdkit: 2025_03_5 (Q1 2025) Release. Zenodo https://doi.org/10.5281/ZENODO.591637 (2025).
